# Syncope and Influenza B: A Case of an Arresting Association

**DOI:** 10.1155/2018/1853473

**Published:** 2018-08-02

**Authors:** Alan Lucerna, James Lee, James Espinosa

**Affiliations:** ^1^Program Director, Combined EM/IM Program, Rowan University SOM/Jefferson Health, Stratford, NJ, USA; ^2^Emergency Medicine, Rowan University SOM/Jefferson Health, Stratford, NJ, USA

## Abstract

Influenza is a contagious viral illness that usually presents with upper respiratory and pulmonary symptoms. While generally self-limited, pulmonary, renal, metabolic, neurologic, and cardiac complications have all been described in the literature. Here we describe a case of a 46-year-old male with multiple episodes of syncope, found to have severe bradycardia, sinus arrest, and positive influenza B, requiring permanent pacemaker placement. The viruses responsible for the flu can be differentiated into four types: A, B, C, and D. The two primary viruses responsible for the seasonal winter epidemic influenza in the United States are Human Influenza A and B viruses. It has been postulated that the influenza virus may be responsible for activating acute inflammatory cytokines, which then alter electrical conduction properties of endothelial cells. Although there have been cases of sinus arrest in association with influenza, some requiring pacemaker placement, our patient's presentation with multiple episodes of syncope with severe bradycardia and sinus arrest requiring permanent pacemaker placement, in association with influenza B, is very unusual and possibly unique. Since emergency physicians are at the forefront in the diagnosis, treatment, and disposition of these patients, awareness of influenza triggered cardiac events is essential and lifesaving, especially in unvaccinated patients.

## 1. Introduction

Influenza is a contagious viral illness which commonly presents with the abrupt onset of fevers, chills, myalgias, and cough. It may often be mistaken for the common cold [1]. Like many viral illnesses, influenza is generally self-limited. However, it can be associated with severe complications, especially in patients with chronic medical conditions, young children, adults aged 65 years and older, and pregnant women [1]. In addition, pulmonary complications are common in patients with the flu. Patients may become more susceptible to developing a secondary bacterial pneumonia [2]. Influenza has been known to cause acute renal failure, rhabdomyolysis [3], acute encephalopathy [4], and cardiac complications such as acute myocarditis [5]. Here we present an unusual case of a patient presenting with multiple syncopal episodes with heart block, requiring a pacemaker, associated with influenza B.

## 2. Case Report

A 46-year-old male presented to the emergency department (ED) after three syncopal episodes. In the preceding three days, the patient experienced the onset of cough, congestion, and generalized malaise. His son had similar symptoms. On the morning of his arrival to the ED, the patient experienced lightheadedness while he was in the bathroom getting ready for work. He lost consciousness and reported waking up against the sink. Twenty minutes later, while sitting on his bed, he experienced another syncopal episode and fell against his dresser. He tried to get up, but again lost consciousness. It was at this time that he called 911 and the patient was brought to the ED. None of the episodes were associated with chest pain, palpitations, shortness of breath, vertigo symptoms, or headaches.

The patient's past medical history was notable for obstructive sleep apnea. He had myringotomy tubes as a child but no other surgeries. He occasionally smoked cigars and drank alcohol socially and had no history of drug use or abuse. He reported that his grandfather had coronary artery disease (CAD). There was no family history of cardiac conduction abnormalities. The patient was allergic to penicillin, latex, and shellfish. He did not take any prescribed or over-the-counter medications.

Vital signs on arrival were blood pressure of 126/70 mm Hg, heart rate of 82 beats per minute, respiratory rate of 16 breaths per minute, and temperature of 99.2 degrees F orally with oxygen saturation of 96% on room air.

The patient was awake and alert in no apparent distress. His head was normocephalic and atraumatic. Neck was supple and the posterior elements did not have any tenderness or step-offs. There were no carotid bruits. The heart exam revealed a normal rate with no murmurs, rubs, or gallops. There was no displacement of point of maximal intensity. The lungs were clear to auscultation bilaterally. Abdomen exam was unremarkable. The extremities had no edema. His skin was warm, dry, without any obvious rashes. The neurological examination was unremarkable.

Diagnostic studies showed serum glucose of 178 mg/dl. Tests of sodium, potassium, BUN, creatinine, TSH, calcium, and magnesium were within normal limits. Cardiac biomarkers were negative. The white blood cell, hemoglobin, hematocrit, and platelet counts were within normal limits.

The chest X-ray did not reveal any evidence of effusions or infiltrates. An influenza swab was influenza B positive. The patient had not received influenza vaccine for the year. A dose of Tamiflu (oseltamivir) was initiated.

**Figure 1:**
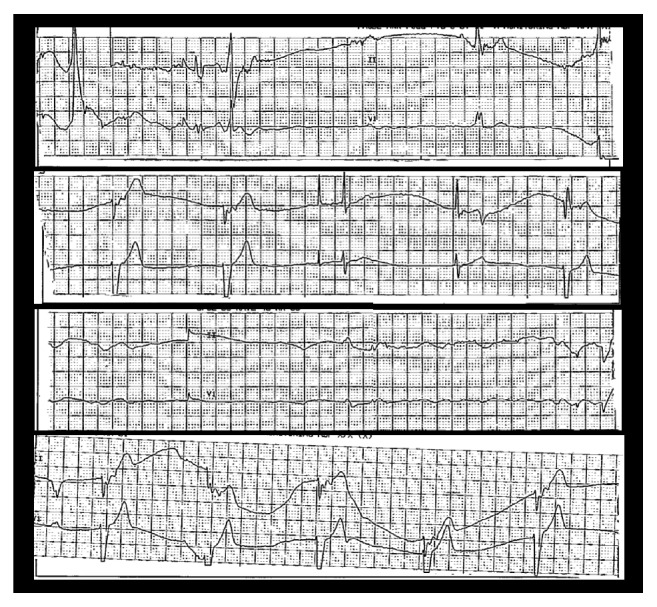
Telemetry strips showing bradycardia and sinus arrest coincidental with the patient's syncope.

While in the ED, the patient had a witnessed syncopal event. Telemetry tracings at the time of the episode was reviewed and showed bradycardia and 6-second sinus arrests [Figure 1].

**Figure 2:**
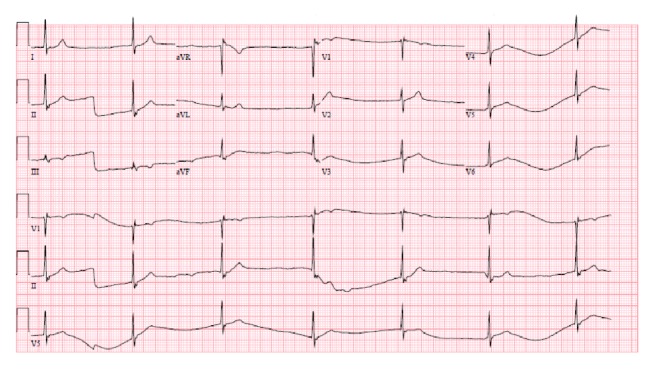
ECG, showing junctional bradycardia.

A 12-lead electrocardiograph (ECG) was obtained and showed junctional bradycardia with a rate of 39 [Figure 2].

The cardiology team evaluated the patient in the ED. A 2D echo showed borderline concentric left ventricular hypertrophy and trace tricuspid regurgitation but preserved left and right ventricular systolic function with ejection fraction estimated to be approximately 60-65%. Given the patient's multiple episodes of syncope, telemetry data, and his ECG findings, a permanent pacemaker was recommended.

**Figure 3:**
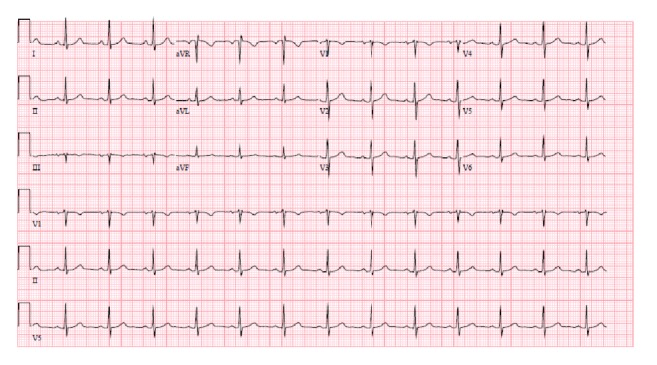
ECG post-op day # 1. Normal sinus rhythm with a rate of 75, and no ischemic changes.

The patient was taken to the OR and a permanent pacemaker was successfully implanted. The patient was monitored in the Intensive Care Unit (ICU) postoperatively. A repeat ECG on post-op day number one showed normal sinus rhythm with rate of about 75 beats per minute and no ischemic changes [Figure 3]. Telemetry monitoring did not show any additional bradycardic episodes or sinus blocks. Lyme IgG and IgM testing was negative. The rest of his hospitalization was unremarkable and patient was discharged to home with an outpatient cardiology follow up one week from discharge.

## 3. Discussion

Influenza is one of the most common viral presentations in the emergency department. It accounts for an estimated annual average of 500 per 100,000 emergency department visits [6] and is responsible for 200,000 hospitalizations annually [1].

The viruses responsible for the flu can be differentiated into four types: A, B, C, and D. The two primary viruses responsible for the seasonal winter epidemic influenza in the United States are Human Influenza A and B viruses. The virus is spread from person to person, primarily via aerosol respiratory droplets, and can be diagnosed by rapid influenza diagnostic tests (RIDT) [1, 7]. Based on surface proteins, influenza A virus is further subdivided whereas influenza B viruses are not differentiated [1]. A common misconception by practicing clinicians is that influenza A tends to have poorer clinical outcomes given its clinical severities and prevalence. However, studies have shown that both viruses were equally virulent and had similar severity. Thus, the specific strain should not guide treatment decisions [1].

Cardiac arrhythmias have been previously reported to occur as complications in patients with the influenza, with the most common finding being acute atrioventricular conduction blocks [8].

Steinberg et al. reported a case of a male with a positive influenza PCR, who presented with episode of presyncope, myalgias, and dyspnea developing over the course of a few days. The patient was evaluated in the hospital and was noted to have a left bundle branch block followed by symptomatic sinus pauses. These symptoms persisted throughout this patient's hospital stay, which resolved with the placement of a permanent pacemaker [9].

Coccagna et al. reported the case of a young male who presented with tachycardia and lightheadedness in association with a positive influenza test. 24-hour Holter monitoring revealed episodes of sinus arrest, which lasted up to 7 seconds. The patient was offered a pacemaker which he declined. His symptoms subsided spontaneously [10].

It has been postulated that the influenza virus may be responsible for activating acute inflammatory cytokines, which then alter electrical conduction properties of endothelial cells [11]. There have been reported findings of influenza RNA in the myocardium of patients with sudden death, which may further suggest that influenza may be responsible for inciting cardiac arrhythmias beyond atrioventricular conduction blocks, such as ventricular fibrillation [8] and cardiac arrest [12].

Cardiac complications secondary to the influenza virus such as myocarditis and pericarditis are well recognized. These cardiac manifestations are typically nonspecific and occur 4 to 9 days after onset of upper respiratory symptoms [13]. Though the precise mechanism remains unclear, there is evidence suggesting that the influenza virus may have a greater role than previously believed in triggering cardiac events, including symptomatic bradycardia and sinus arrest [8]. The rates of cardiac complications secondary to influenza are 15% to 43% in patients who are ambulatory and 14% to 75% in patients who are hospitalized [14].

Although there have been cases of sinus arrest in association with influenza, some requiring pacemaker placement, the patient's presentation with multiple episodes of syncope with severe bradycardia and sinus arrest requiring permanent pacemaker placement, in association with influenza B, is very unusual and possibly unique. It is noteworthy that the patient had not received an influenza vaccination in the year of his illness. It is possible that influenza vaccination may have prevented his illness.

## 4. Conclusion

Influenza while is generally self-limited, our case describes a patient with multiple episodes of syncope, found to have severe bradycardia, sinus arrest, and positive influenza B, requiring a pacemaker placement. It has been postulated that the influenza virus may be responsible for activating acute inflammatory cytokines that can alter electrical conduction properties of cardiac endothelial cells. Although there have been cases of sinus arrest in association with influenza, some requiring pacemaker placement, our patient's presentation with multiple episodes of syncope with severe bradycardia and sinus arrest requiring pacemaker placement, in association with influenza B, is possibly unique. Since emergency physicians are at the forefront in the diagnosis, treatment, and disposition of these patients, awareness that influenza can have a greater role in triggering cardiac events is essential and lifesaving especially in unvaccinated patients.
